# Activation-associated death of memory b cells in peripheral circulation in adults with sepsis

**DOI:** 10.1186/2197-425X-3-S1-A621

**Published:** 2015-10-01

**Authors:** M Shankar-Hari, R Beale, M Singer, J Spencer

**Affiliations:** Department of Immunobiology, King's College London, London, United Kingdom; Guy's and St Thomas' NHS Foundation Trust, Critical Care Medicine, London, United Kingdom; University College London, Intensive Care Medicine, London, United Kingdom; Research Department of Clinical Physiology, Division of Medicine, University College London, London, United Kingdom

## Introduction

In sepsis, impaired function and loss of antigen-presenting cells are observed in secondary lymphoid organs [1], the site where antigen-dependent B cell differentiation occurs in health. How these changes in sepsis affect B cell differentiation into memory B cells is at present undefined.

## Objectives

To study seven-day lymphocyte and immunoglobulin trajectory, alterations in B cell subsets and potential mechanisms in septic ICU patients.

## Methods

Adults with severe sepsis from community-acquired infections without documented immunosuppression were enrolled. Hypogammaglobulinaemia and absolute lymphopenia were defined as IgG < 6.1, IgM < 0.4, IgA < 0.8 g/L and lymphocytes < 1.2 × 10^9^/L, respectively.

Flow cytometry [FACScalibur [BD Biosciences]; FlowJo software,] was used for:

Identifying naïve, transitional, IgM, IgG and IgA memory and plasmablasts using anti-human CD19-PerCpCy5.5, IgG-APC H7, IgM-V450, CD24-PeCy7 [all BD Biosciences], IgA-FITC, CD38-PE, Annexin V Apoptosis detection set PE-Cy7 [all eBioscience] and live-dead stain [Invitrogen]. ADDIN EN.CITE ADDIN EN.CITE.DATA [2], [3]

Intracellular staining to assess phosphorylated kinase expression in B cells [p-ERK-PE, p-BTK-alexafluor 647, p-SYK-alexafluor 488, p-AKT-APC [all BD Biosciences].

FMO and isotype controls were used to define population gates.

B cell survival ligands [BAFF, APRIL] were measured using ELISA.

Differentially expressed genes in sepsis are reported [RT-q-PCR, TaqMan^®^ Human Apoptosis Array; false discovery rate = 5%].

Statistics were performed using paired and unpaired t test or non-parametric equivalent with adjustment for collinear measurements.

## Results

101 patients were studied. On their first ICU day, 46% had hypogammaglobulinaemia and 76% absolute lymphopenia with absolute low B [75%] and T [100%] lymphocyte counts. Trajectory of significantly higher increment immunoglobulins and lymphocyte counts occur earlier in survivors compared to non-survivors.

In sepsis [compared to healthy controls, n=variable] there was

Preferential apoptotic loss of memory B cells and plasmablasts, with apoptotic cells showing higher phosphorylated extracellular signal-regulated kinases [p-ERK fluorescence], but no differences in the phosphorylated B cell receptor linked kinases [p-BTK, p-SYK] and protein kinase B.

BAFF/APRIL levels were normal.

Fas and bcl-2 apoptosis regulator genes were up regulated.

## Conclusions

In sepsis, activation-associated B cell apoptosis and changes in secondary lymphoid organs deplete B cell memory and contribute to long-term immunosuppression in survivors.

## Grant Acknowledgment

UK NIHR, Biomedical Research Centre at Guy´s and St Thomas´ NHS Trust & King´s College London
